# Aerobic exercise for substance use disorders: therapeutic effects and underlying mechanisms

**DOI:** 10.3389/fmed.2026.1915814

**Published:** 2026-08-19

**Authors:** Qiao-qiao Liu, Xiao-xuan Zheng, Shang-wen Wang, Lin Miao, Ying-jie Zhao, Jian-xing Liu, Juan Li, Jian Huang

**Affiliations:** 1 Department of Pathogen Biology and Immunology, School of Basic Medical Science, Kunming Medical University, Kunming, China; 2 National Health Commission (NHC) Key Laboratory of Drug Addiction Medicine, School of Forensic Medicine, Kunming Medical University, Kunming, China

**Keywords:** addictive behavior, aerobic exercise, neural mechanisms, relapse intervention, substance use disorder

## Abstract

Substance use disorder (SUD) is a psychiatric condition in which recurrent use of psychoactive substances, including alcohol, nicotine, opioids, and stimulants, induces pathological alterations in brain rewards, motivation, and memory circuitry, leading to loss of control over use, compulsive drug-seeking behavior, heightened tolerance, and significant impairment in social functioning. SUD should not be construed as a moral failing or a volitional weakness, but rather as a chronic, relapsing brain disorder. Dependence on tobacco, alcohol, and illicit substances such as opioids and amphetamines constitutes distinct forms of SUD, marked by frequent relapse and associated with profound societal harm and an escalating global public health threat. Although pharmacotherapy and psychosocial interventions remain the mainstay of SUD treatment and can reduce withdrawal symptoms and craving, long-term outcomes are often disappointing. Pharmacotherapy is limited by side effects and high dropout rates, while psychosocial treatments are hampered by poor adherence and uneven access to care–factors that help account for the persistently high relapse rates seen in clinical practice. In contrast, exercise is a safe, non-pharmacological add-on treatment that modulates brain function and alleviates psychiatric and cognitive symptoms associated with addiction. These effects can reduce drug craving, curb compulsive use, and interfere with relapse-promoting processes, offering measurable clinical benefit. In this review, we summarize the effects of exercise interventions on individuals with drug, alcohol, and tobacco addiction. We discuss the potential mechanisms behind these benefits, highlight current limitations in the field, and suggest directions for future research. This review aims to support the development of new intervention strategies and to synthesize the current evidence on the therapeutic effects and mechanistic basis of aerobic exercise in SUD.

## 1 Introduction

Substance use disorder (SUD) is a chronic, relapsing brain disease characterized by prolonged, repeated use of psychoactive substances such as alcohol, tobacco, and illicit drugs (1), which results in loss of control over use, intense craving, withdrawal symptoms, and broad functional impairment (2, 3). According to the World Drug Report 2025, an estimated 316 million people worldwide used drugs in the past year, and approximately 64 million suffered from drug use disorders.

Cannabis use disorder exhibits the highest prevalence but the lowest disease burden, whereas opioid use disorder carries the heaviest burden and constitutes the primary source of disease burden. Although amphetamine use disorder is declining globally, its prevalence remains elevated in high-income countries and regions impacted by cannabis legalization, where it demonstrates significant comorbidity with opioid use disorder (4). Alcohol use disorder (AUD) affects approximately 400 million people worldwide and remains the third leading cause of disease burden attributable to mental disorders globally (5). Tobacco use continues to be a major cause of death and disability and is a key risk factor for cardiovascular and cerebrovascular diseases, chronic obstructive pulmonary disease, and multiple cancers (6, 7).

High relapse rates continue to pose a significant challenge in the treatment of SUD. Traditional psychosocial interventions, such as cognitive behavioral therapy, motivational interviewing, and contingency management, are effective and increasingly used in community settings, though their reach is often constrained by limited professional resources, training demands, and regional disparities (8, 9). Thus, there is an urgent need for safe, cost-effective, and scalable non-pharmacological strategies. In recent years, aerobic exercise has gained increasing attention as an important intervention, owing to its favorable safety profile and potential therapeutic benefits. This review provides a systematic overview of intervention research on aerobic exercise in individuals with drug, tobacco, and alcohol addiction, and further attempts to synthesize the existing evidence and propose directions for future research.

## 2 Aerobic exercise

Aerobic exercise, also referred to as endurance exercise, is typically characterized by the rhythmic and sustained engagement of the body’s large muscle groups. Common forms of aerobic exercise include brisk walking, running, swimming, and cycling, among others. Aerobic exercise can be classified, according to intensity, into moderate-intensity and high-intensity categories. Moderate-intensity aerobic exercise is characterized by a moderate increase in heart rate and breathing, yet the individual can still carry on a normal conversation; a weekly total of 150–300 min is recommended for adults and older adults. High-intensity aerobic exercise, on the other hand, is marked by a pronounced increase in heart rate and breathing that makes comfortable conversation difficult, with a recommended weekly total of 75–150 min. The two intensity levels can be combined and converted at an approximate ratio of 1:2 (e.g., 1 min of high-intensity exercise is generally regarded as equivalent to 2 min of moderate-intensity exercise), thereby accommodating the varying fitness levels and health needs of different populations (10). Multiple studies have demonstrated that aerobic exercise confers broad benefits for physical and mental health across diverse populations, including slowing brain aging in healthy adults (11), improving metabolic and inflammatory profiles in older adults (12), markedly improving quality of life in women diagnosed with breast cancers during chemotherapy treatment (13), and alleviating depressive and anxiety symptoms in children, adolescents, and adults (14, 15).

Beyond its established benefits for a range of medical conditions, aerobic exercise is increasingly recognized for its therapeutic potential for SUD. Regular aerobic exercise has been shown to enhance prefrontal cortex activity and cognitive function, strengthening impulse control and decision-making in individuals with SUD (16, 17), while simultaneously modulating the striatal dopamine rewards system to reduce craving for addictive substances (18, 19). Through these mechanisms, aerobic exercise effectively improves impulse control (20), emotional state (21), and drug craving (22) in individuals with SUD. Moreover, this intervention offers a favorable safety profile, minimal side effects, and no risk of dependence, while also contributing to improved cardiometabolic health and a reduction in associated somatic comorbidities (23, 24).

## 3 Intervention effects of aerobic exercise on SUD

### 3.1 Intervention effects of aerobic exercise on individuals with drug addiction

According to their historical emergence and characteristic features, drugs are divided into traditional and novel types. Traditional drugs are typically natural or semi-synthetic substances, such as heroin, cannabis, and cocaine, that emerged earlier and have a long history of addiction. In contrast, novel drugs are primarily synthetic psychoactive substances, including methamphetamine, MDMA (ecstasy), ketamine, and magu (a methamphetamine-caffeine mixture). These substances are often disguised as other products or marketed under different names, making them difficult to identify and regulate. The benefits of aerobic exercise for individuals with drug addiction are mainly manifested in reducing cravings, alleviating anxiety and depression, improving cognitive function and self-control, modulating neuroendocrine function, and repairing oxidative stress damage.

In traditional drug addiction, intense drug craving, high relapse risk, and marked emotional disturbances are frequently observed, along with cognitive impairment and neuroendocrine dysregulation in some patients, all of which are critical factors contributing to unsuccessful withdrawal management and relapse (25–27). In a study of 59 adult patients with moderate - to - severe cannabis use disorder, a 12-week intervention consisting of either high-intensity interval training or resistance training did not yield significant improvements in hippocampal measures, cognitive function, or mood disturbance. However, both exercise modalities significantly reduced cannabis craving (28). Similarly, a study of 90 patients with opioid dependence found that those receiving opioid substitution therapy who engaged in a 2-month aerobic exercise program showed a significant reduction in weekly cocaine use days, with the effect becoming more pronounced after the fifth week of the intervention (29). Further research has found that the mood-improving effects of aerobic exercise are particularly notable in individuals with traditional drug addiction. Multiple studies conducted among heroin-dependent individuals have confirmed that aerobic exercise can improve addiction-related psychological and cognitive functions. Notably, moderate-intensity aerobic exercise has been shown to significantly reduce drug craving and relapse risk in heroin-dependent individuals, while also alleviating negative emotional states such as anxiety and depression (30). Similarly, high-intensity aerobic exercise and resistance training also reduce drug craving, enhance positive emotions, attenuate attentional bias toward drug-related cues, and improve inhibitory control and working memory (31). Furthermore, aerobic exercise can also aid in abstinence through modulation of neuroendocrine function. A study of 90 adult patients with opioid use disorder who were receiving substitution therapy assigned them to either an aerobic exercise group (8 weeks of moderate-intensity aerobic exercise, 3 times per week, 20 min per session, at 70% of maximum heart rate) or a non-exercising control group. β-Endorphin levels increased significantly and cortisol levels decreased markedly in the aerobic exercise group (32). This restoration of neuroendocrine homeostasis contributes to alleviating withdrawal symptoms, improving emotional regulation, and reducing relapse risk. In summary, aerobic exercise has demonstrated a range of beneficial effects in interventions for traditional drug addiction, primarily through reducing craving, improving emotion and cognition, and modulating neuroendocrine function.

Among novel drugs, methamphetamine is the most widely studied and commonly used representative of this category. We therefore focus on methamphetamine here to illustrate the effects of aerobic exercise on novel drug addiction, given its public health significance and the relative abundance of available evidence. Methamphetamine abuse commonly leads to oxidative stress, cognitive impairment, reduced executive function, and autonomic nervous system dysregulation. These deficits not only exacerbate addictive behaviors but also compromise post-withdrawal recovery outcomes (33, 34). In methamphetamine use disorder (MUD), a 12-week program of moderate-intensity aerobic exercise significantly attenuates the spontaneous increase in serum malondialdehyde (a marker of oxidative stress) during withdrawal and improves cognitive function (35). In another study of acute aerobic exercise in patients with MUD, a single 30-min session of moderate-intensity aerobic exercise, versus a 30-min non-exercise reading control, significantly reduced the craving for methamphetamine, improved inhibitory control over responses to drug-related cues, and enhanced electrophysiological activity associated with conflict monitoring (increased N2 amplitude) (36). These findings suggest that acute aerobic exercise offers clear benefits for self-control and craving reduction in individuals with addiction. Long-term aerobic exercise produces significant and sustained improvements in cognitive function among individuals with MUD. A randomized controlled trial involving 89 female patients with MUD demonstrated that a 12-week program of supervised, moderate-intensity aerobic exercise significantly improved attention, working memory, and executive function, with improvements sustained for up to 3 months post-intervention (37). Another study involving 330 patients with MUD confirmed that an 8-week intervention of moderate-intensity aerobic exercise (5 sessions per week, 60 min per session, treadmill walking or jogging) significantly improved autonomic nervous system function, cardiorespiratory fitness, and executive function (38). Overall, aerobic exercise is of considerable importance for both physical and cognitive rehabilitation for individuals with methamphetamine addiction and may serve as a valuable adjunct to withdrawal and recovery.

### 3.2 Intervention effects of aerobic exercise on individuals with tobacco addiction

Tobacco addiction is clinically diagnosed as nicotine use disorder (NUD), which is a type of SUD. Thus, individuals with tobacco addiction are essentially individuals with NUD. During nicotine withdrawal, individuals frequently experience a cluster of adverse symptoms, including anxiety, irritability, impaired concentration, and craving. These negative emotional states, together with the persistent urge to smoke, are key factors that contribute to smoking relapse (39–41).

In interventions for NUD, aerobic exercise has shown beneficial effects in alleviating smoking craving and withdrawal symptoms, and significantly improves mood in individuals with NUD (42). Focusing on individuals with NUD and comorbid depressive symptoms, one study examined the effects of a 12-week moderate-intensity aerobic exercise program on mood and smoking craving. The data showed that a single bout of moderate-intensity aerobic exercise acutely reduced smoking craving. Following the long-term intervention, although no sustained improvements were observed in baseline mood or craving levels, the exercise group continued to exhibit a significant decline in craving immediately after each exercise session. Further analysis revealed that, within the exercise group, higher levels of positive affect were associated with lower craving, suggesting that positive affect may mediate the effect of exercise on smoking craving (43).

Of note, however, aerobic exercise has not yielded pronounced long-term effects in individuals with NUD. A meta-analysis showed that long-term aerobic exercise interventions were no more effective than health education or sedentary control conditions in improving smoking cessation rates. However, acute aerobic exercise was effective in reducing smoking craving and negative mood (44). In addition, chronic smoking leads to executive dysfunction, manifesting as cognitive deficits that include diminished attention and impaired response inhibition. Evidence indicates that both regular, long-term aerobic exercise and a single bout of acute aerobic exercise can ameliorate these executive function deficits and modulate brain activation patterns (45).

Although some evidence indicates that aerobic exercise may not directly help individuals addicted to tobacco or e-cigarettes achieve complete abstinence, its protective and restorative effects on the body remain substantial (46). Aerobic exercise can mitigate cigarette smoke-induced lung injury, preserve pulmonary function, reduce oxidative stress, and modulate the expression of antioxidant and anti-inflammatory factors (47) and can reverse extrapulmonary impairments caused by long-term smoking, such as early-stage cardiac damage and peripheral vascular endothelial dysfunction (48). Overall, aerobic exercise substantially improves overall health in individuals with NUD, with particularly marked benefits for cardiorespiratory function, and positively affects psychological well-being. Although its long-term effects on smoking cessation itself remain uncertain, its acute effects on craving reduction and its overall health benefits support its use as a complementary strategy in tobacco addiction management.

### 3.3 Intervention effects of aerobic exercise on individuals with alcohol addiction

The core clinical features of AUD are compulsive alcohol use and diminished control over drinking, with typical symptoms including intense alcohol craving and a withdrawal syndrome after cessation (e.g., tremor and insomnia) (49, 50). A clinical study using self-report questionnaires examined the effects of a single bout of moderate-intensity aerobic exercise on alcohol craving. The results showed that alcohol craving decreased significantly after exercise, with the majority of participants experiencing relief and nearly half showing a clinically meaningful improvement (51). However, there was considerable interindividual variability, with a minority of participants showing no meaningful change in alcohol craving or even an increase in craving. Further analysis indicated that individuals with higher pre-exercise alcohol craving and relatively poorer cardiorespiratory fitness were more likely to benefit from a single bout of aerobic exercise. A meta-analysis of individuals with AUD found that aerobic exercise interventions did not significantly improve weekly alcohol consumption; however, they significantly reduced daily alcohol intake and lowered scores on the Alcohol Use Disorders Identification Test (AUDIT) (52).

Nevertheless, the available evidence has not consistently shown that exercise interventions significantly reduce alcohol consumption among individuals with AUD. For example, one study assigned individuals with AUD to an experimental group that received supervised group and individual exercise, and a control group that received treatment as usual, and compared alcohol consumption before and after the intervention (e.g., proportion of heavy drinking days, drinking frequency, and daily intake). The results revealed no statistically significant difference in alcohol consumption between the exercise intervention group and the control group (53). In addition, studies have shown that aerobic exercise significantly improves anxiety, depression, and stress levels in individuals with AUD (54). Furthermore, individuals with AUD often have comorbid hepatic dysfunction, such as fatty liver disease and cirrhosis, as well as cardiovascular impairment, including hypertension (52, 55). Aerobic exercise is particularly beneficial in AUD patients with metabolic disturbances by attenuating hepatic injury and oxidative stress (56). Additionally, aerobic exercise helps improve physical fitness and cardiovascular health, thus enhancing overall physical function in these patients (57). Thus, incorporating exercise as a clinical adjunctive intervention may help improve quality of life and reduce the likelihood of relapse in individuals with AUD.

## 4 Core mechanisms of aerobic exercise interventions in SUD

The core pathology of SUD is characterized by dysfunction of the central nervous system. Among these, the aberrant activation and dysfunction of the brain’s rewards system are a key driver of the initiation, progression, and relapse that define addiction (58, 59). The mesolimbic dopamine system, which is central to the rewards system, is primarily composed of the ventral tegmental area (VTA), nucleus accumbens (NAc), dorsal striatum (DS, encompassing the caudate putamen, CPu), and prefrontal cortex (PFC). It modulates rewards perception, motivational drive, and behavioral reinforcement through dopaminergic neurotransmission (60–62). In normal physiological conditions, this system maintains adaptive responses to natural rewards such as food and exercise. However, addictive substances disrupt this homeostatic balance and drive the formation of pathological rewards memories. Aerobic exercise can modulate central nervous system function via multiple targets and pathways, thereby ameliorating the diverse pathological impairments associated with SUD. In the following sections, we detail how aerobic exercise reshapes the rewards system, enhances cognitive control, and restores physiological and emotional homeostasis.

### 4.1 Rewards system remodeling

Dysregulation of the rewards system is a core feature of addiction, making it a key target for intervention. Aerobic exercise bidirectionally modulates dopamine receptors in the mesolimbic system by reducing D1 receptor and increasing D2/D3 receptor expression, thus restoring rewards circuit homeostasis. In patients with MUD, an 8-week aerobic exercise training program significantly increased striatal D2/D3 receptor availability, suggesting that structured exercise can ameliorate these deficits and warrant further evaluation as an adjunctive treatment for stimulant dependence (63). In animal studies, 6 weeks of treadmill training significantly decreased D1 receptor binding in the nucleus accumbens shell and increased D2 receptor binding in the core. Similar D2 increases were observed in the dorsomedial, ventrolateral, and ventromedial caudate putamen, as well as in the olfactory tubercle (64), suggesting that aerobic exercise may reduce drug-seeking behavior through dopamine receptor modulation. Beyond this, aerobic exercise can counter addiction by activation other neural circuits. Long-term wheel running markedly increases c-fos expression in the magnocellular part of the red nucleus (RNm). Glutamatergic projections from the RNm to the VTA enhance synaptic transmission, and activating this pathway mimics the rewards effect of exercise and suppresses cocaine addiction in mice (65). In a rat model of methamphetamine withdrawal, dopamine neurons abnormally increase in the periaqueductal gray (PAG), and aerobic exercise prevents this, reducing subsequent drug-seeking and relapse (66). At the behavioral level, the intrinsic non-drug rewards properties of aerobic exercise can reduce or compete with the reinforcing effects of substance use, thus helping to suppress drug-seeking behavior and relapse risk. This effect goes beyond simple behavioral substitution and likely contributes critically to the adjunctive treatment of drug dependence by promoting neuroplastic changes that run counter to those induced by addiction (67). Animal imaging studies have shown that long-term regular aerobic exercise significantly reshapes brain glucose metabolic response patterns during chronic cocaine exposure (68). In core brain regions closely implicated in rewards encoding, emotion regulation, and addictive behavior, including the bed nucleus of the stria terminalis, caudate putamen, and thalamus, exercise intervention exerted metabolic inhibitory effects (68). These brain regions are integral components of the mesolimbic-mesocortical rewards pathway and are directly involved in encoding the emotional salience of rewards stimuli, regulating stress-related responses, and driving cocaine-seeking behavior (68).

The mesolimbic dopaminergic pathway is the core neural substrate of alcohol rewards, and exercise and alcohol compete through a “hedonic substitution” mechanism within this pathway. In animal models, moderate-intensity exercise can attenuate alcohol-induced neuronal damage, an effect closely tied to exercise-induced remodeling of the rewards pathway (69). Chronic ethanol dependence leads to both upregulation and functional sensitization of kappa opioid receptors (KOR) in the NAc of mice. Voluntary wheel-running exercise downregulates KOR expression and alters KOR sensitivity of dopamine release in the same brain area, ultimately reducing subsequent ethanol intake (70). This suggests that exercise achieves a “functional substitution” for alcohol-use behavior through opioid receptor-mediated remodeling of the rewards pathway. The ameliorative effects of exercise on smoking craving and withdrawal symptoms are largely mediated by its favorable modulation of the mesolimbic dopamine pathway. As with alcohol, the rewards substitution effect represents a key mechanism by which exercise promotes smoking cessation. Taken together, aerobic exercise systematically counteracts the rewards system dysfunction induced by substance addiction, at multiple levels from receptor balance and circuit remodeling to behavioral substitution.

### 4.2 Enhancement of cognitive control

Cognitive impairment is common in SUD and predicts poor treatment outcomes. Improving cognitive control is therefore clinically relevant. Aerobic exercise can significantly improve cognitive control in individuals with SUD by enhancing neuroplasticity. A 3-month randomized controlled trial confirmed that aerobic exercise significantly improved multiple cognitive functions in women with methamphetamine dependence, including attention, working memory, delayed verbal memory, and executive function, with improvements attributed to exercise-induced enhancement of prefrontal cortical activity. Furthermore, the beneficial effects remained stable for up to 3 months after the intervention and were markedly superior to those achieved with usual care alone (37). Multiple animal studies have confirmed that aerobic exercise provides clear neuroprotective and cognitive restorative effects against SUD-induced brain injury. In morphine-addicted rats, aerobic exercise ameliorates cognitive dysfunction and anxiety-like behavior. Mechanistically, these benefits may involve attenuation of hippocampal oxidative stress and inflammatory injury, as well as restoration of synaptic plasticity (71). In methamphetamine-addicted mice, treadmill exercise increases expression of postsynaptic density protein 95 (PSD-95), modulates key signaling pathways (PI3K-Akt, mTOR, and Wnt), and regulates gene expression (e.g., upregulating NFKBIA to suppress neuroinflammation while improving CXCL12- and Vav3-related neuronal function). Together, these actions inhibit neuroinflammation, restore synaptic plasticity, and improve learning and memory deficits (72). In mice with AUD, daily wheel running suppresses the escalation of alcohol consumption, an effect associated with increased brain-derived neurotrophic factor (BDNF) expression in the medial prefrontal cortex and dentate gyrus, as well as enhanced BDNF-TrkB signaling (73). Furthermore, treadmill training also significantly ameliorates alcohol-induced impairments in complex motor performance and improves motor learning deficits in mice (74). A core cognitive feature of nicotine addiction is smokers’ approach bias toward tobacco-related cues. Research has shown that smokers exhibit a marked approach bias toward tobacco-related cues, and the magnitude of this bias is positively correlated with the level of nicotine dependence. Acute aerobic exercise can temporarily enhance cognitive control over smoking-related cues, attenuate approach bias, and improve prefrontal cortex function in smokers (75).

Moreover, aerobic exercise can enhance cognition through synergistic actions across multiple targets and pathways. In the context of morphine-induced brain injury in rats, aerobic exercise upregulates the expression of BDNF, SIRT3, and SIRT4, suppresses aberrant neuroinflammation and oxidative stress, and promotes the recovery of neuronal function (71). In addition, aerobic exercise mitigates methamphetamine-induced oxidative stress in mice and counteracts its neurotoxicity (76), effects that may benefit cognitive function. According to a review by Zhai and colleagues, aerobic exercise stimulates irisin release, enabling it to cross the blood–brain barrier and act on the prefrontal cortex, where it restores executive function in individuals with SUD by upregulating BDNF, attenuating neuroinflammation, and strengthening synaptic plasticity, thereby reducing relapse risk (77). In summary, findings from preclinical studies indicate that aerobic exercise enhances neuroplasticity in the prefrontal and motor cortices, and ameliorates deficits in cognitive control and behavioral regulation associated with substance addiction. These mechanistic insights are primarily derived from animal models, whereas human studies have largely focused on the functional outcomes of exercise interventions, such as improvements in cognitive performance. Future research integrating neuroimaging with behavioral measures in human subjects would help to bridge this gap and further validate the neurobiological pathways suggested by animal work.

### 4.3 Restoration of physiological and emotional homeostasis

Negative affect during withdrawal is a major driver of relapse, making the stabilization of emotional and physiological states clinically relevant. Prolonged substance abuse induces compensatory changes in the central dopamine system, resulting in a pathological disruption of neural homeostasis that manifests as anxiety and depression during withdrawal. As a multisystem physiological stimulus, exercise offers a unique restorative effect on emotional homeostasis by broadly activating rewards pathways, re-establishing neurotransmitter homeostasis, and remodeling stress-regulatory centers.

In a 6-week adjunctive exercise program combined with standard intensive outpatient treatment for SUD patients, a single bout of aerobic exercise acutely increased anandamide (AEA) levels, enhanced vigor, and reduced negative mood states (tension, depression, anger, confusion) and craving (78). Aerobic exercise also suppresses microglial activation, modulates central and peripheral inflammatory responses, creates a supportive microenvironment for neuronal recovery, and alleviates methamphetamine-induced anxiety-like symptoms in mice (79). This finding demonstrates that exercise relieves anxiety by modulating inflammatory responses rather than substituting rewards signals, providing neuroimmunological evidence for its adjunctive role in addiction treatment. Furthermore, aerobic exercise effectively modulates metabolic dysregulation in the brains of methamphetamine-addicted mice, specifically addressing abnormalities in glycerophospholipid metabolism, steroid hormone biosynthesis and degradation, and the renin-angiotensin system (RAS) pathways. These modulatory effects are thought to help maintain blood-brain barrier integrity, preserve neuronal function, and ultimately alleviate methamphetamine-induced central nervous system injury (80). Moderate-intensity aerobic exercise interventions can significantly improve cardiorespiratory fitness, muscular strength, and overall physical performance in individuals with methamphetamine addiction (81). These positive physiological changes not only directly help mitigate the systemic health complications arising from substance abuse, but may also provide a crucial physiological and neurobiological foundation for reducing the risk of relapse, through improvements in brain health such as optimized cerebral perfusion and repair of the damaged nervous system. According to a review, aerobic exercise can restore the balance between interoception and physiological homeostasis by calibrating bodily signals, regulating hormonal levels, and optimizing cardiometabolic function, while also modulating brain networks centered on the insular cortex. Through these improvements, it enhances organismal homeostasis and contributes to long-term recovery in individuals with SUD (82).

Prolonged alcohol exposure and withdrawal readily precipitate negative emotional states, and exercise shows considerable promise in this context. For example, Liu et al. (54) conducted a systematic review and meta-analysis of 15 randomized controlled trials and found that exercise interventions significantly improved mental health outcomes in individuals with AUD, with marked reductions in self-reported anxiety symptoms, depressive symptoms, and stress. Interventions lasting less than 14 weeks were more effective in alleviating anxiety and depression. Nicotine withdrawal is likewise characterized by negative affective symptoms, including anxiety, depression, and irritability. Park et al. (83) established a nicotine withdrawal model (subcutaneous nicotine, 6 mg/kg, for 17 consecutive days, followed by 2 weeks of withdrawal) and observed that rats in withdrawal exhibited reduced locomotor activity and pronounced anxiety-like behavior. Treadmill exercise (31 days, 5 days per week, once daily) significantly increased activity levels and alleviated anxiety in these rats. Further mechanistic investigation revealed that the expression of tryptophan hydroxylase and serotonin in the dorsal raphe was decreased in withdrawn rats, and treadmill exercise markedly reversed these changes. Furthermore, Zaniewska et al. (84) demonstrated that wheel-running exercise induced a marked antidepressant effect on day 14 of nicotine withdrawal in a rat model, an effect closely linked to increased hippocampal neurogenesis. This finding provides direct evidence of neuroplasticity for the exercise-induced improvement of depressive symptoms during nicotine withdrawal.

## 5 Conclusions and future directions

### 5.1 Overview of benefits

As a non-pharmacological intervention, aerobic exercise offers several advantages in the prevention and treatment of SUD. First, it exerts its multi-target effects by simultaneously promoting neural circuit remodeling, ameliorating psychological symptoms, and repairing physiological damage, thus bypassing the limitations of pharmacotherapy alone (85, 86). Second, it has a favorable safety profile and carries no risk of dependence, making it well-suited as a long-term maintenance treatment strategy (87, 88). Third, its strong potential for widespread dissemination, given its relatively low cost and diverse modalities, facilitates implementation as an adjunctive therapy in community and rehabilitation settings (86). Current evidence shows that aerobic exercise exerts beneficial effects across various types of SUD, although the strength of evidence varies by substance and by clinical outcome. In methamphetamine addiction, aerobic exercise reduces craving and relapse risk, and promotes neural function recovery (85). In alcohol addiction, exercise improves quality of life and reduces relapse risk (52). In tobacco addiction, the evidence is more mixed: although long-term effects on cessation rates are limited, acute aerobic exercise consistently alleviates craving and withdrawal symptoms (42, 44). These effects operate at multiple levels, including rewards system remodeling, neurotransmitter modulation, inflammation suppression, and psychological and behavioral improvement, thereby providing a theoretical foundation for the clinical application of aerobic exercise in SUD intervention.

### 5.2 Limitations

However, several limitations remain in the current research. First, exercise protocols have yet to be standardized across existing studies. The variability in exercise type, intensity, frequency, and duration across studies has resulted in a lack of standardized intervention protocols and well-defined dose-response relationships. This hinders the establishment of standardized protocols and the evaluation of specific regimens and their associated biomarkers. Moreover, the extent to which different exercise intensities ameliorate withdrawal symptoms varies substantially. Existing evidence indicates that moderate-intensity exercise is most effective in alleviating depression and anxiety, whereas high-intensity exercise yields the most pronounced relief of the overall withdrawal syndrome; for symptoms such as substance craving, stress, irritability, and agitation, the effects of different exercise intensities do not differ significantly. Furthermore, the effects of different aerobic exercise intensities also show a degree of specificity depending on the type of SUD. In AUD, moderate-intensity exercise is preferred for alleviating negative affective states such as depression and anxiety. For opioid use disorder, high-intensity exercise is more effective at alleviating somatic withdrawal symptoms. For psychostimulant use disorders involving methamphetamine and cocaine, both moderate- and high-intensity exercise can effectively ameliorate withdrawal-related symptoms, with high-intensity exercise producing greater craving reduction (89). Second, the effects of exercise interventions on addiction exhibit substantial interindividual variability, and their efficacy is influenced by a range of factors, including sex, age, and the specific type of addiction. Among these, sex, as an important moderating variable, shows marked sex differences in the development, progression, and treatment responsiveness of addiction. Research has demonstrated that women differ markedly from men throughout the entire course of addiction, including its initiation, rate of progression, and response to the reinforcing effects of drugs; they are not only more vulnerable to the adverse consequences of addiction, but also more sensitive to the positive subjective effects of drugs (90). Specifically, among individuals who abuse drugs, including methamphetamine, women show a higher susceptibility to relapse and a more rapid escalation of addiction, a phenomenon widely believed to be closely linked to the modulatory effects of estrogen on the dopamine system (91). Notably, although exercise interventions have been widely recognized as an effective strategy for addiction treatment, emerging evidence suggests that women may require a greater volume of exercise than men to achieve comparable therapeutic benefits. This highlights possible sex-dependent differences in intervention outcomes and underscores the challenges that may arise when applying exercise interventions across sex groups (92). Different addiction types also show distinct responses to exercise intensity; for example, moderate-intensity exercise is sufficient to relieve tobacco craving, whereas higher-intensity exercise may be needed to reduce opioid craving (93). Third, evidence regarding the long-term effects of exercise remains limited. Most studies have included follow-up periods of less than 1 year, resulting in a paucity of robust evidence on the effects of exercise interventions on medium- and long-term relapse rates in individuals with SUD; future follow-up studies are needed to provide more reliable data on the efficacy of exercise. Finally, the mechanisms underlying the therapeutic effects of exercise in SUD remain poorly understood. Although peripheral stress biomarkers have been extensively studied, current studies are limited by methodological shortcomings in elucidating the long-term effects of exercise, the specific regulatory pathways through which it modulates stress responses, and the mediating mechanisms that link exercise to cognitive improvements. Future research should prioritize rigorously designed, longitudinal, multilevel intervention studies with controlled experimental conditions to validate the stress-mediated mechanisms linking exercise to brain health (94). Although existing studies have attempted to integrate molecular and brain structural measures, direct evidence for a causal chain linking exercise, molecular changes, brain function, and behavioral outcomes remains largely absent, and the corresponding mediating effects have yet to be adequately validated (95). This has thus become a major bottleneck in the mechanistic research on aerobic exercise that urgently awaits resolution.

Despite these limitations, the evidence reviewed here supports several clinically relevant directions. For patients with alcohol or nicotine use disorders, moderate-intensity exercise appears to be a reasonable first-line option for improving mood and reducing craving, whereas higher-intensity exercise may offer additional benefit for somatic withdrawal symptoms in opioid dependence and for craving reduction in stimulant users. In practice, exercise prescriptions should be individualized based on the type of substance used, the phase of recovery (acute withdrawal vs. long-term maintenance), and the patient’s baseline fitness level. Rather than advocating a one-size-fits-all approach, we suggest that the dose and modality of exercise be titrated against symptom response and tolerability, similar to how pharmacotherapies are adjusted in clinical settings. While further research is needed to refine these parameters, the existing evidence is sufficient to justify integrating aerobic exercise into addiction treatment protocols as an adjunctive, low-risk, and broadly accessible intervention.
